# Pavement Quality Evaluation Using Connected Vehicle Data

**DOI:** 10.3390/s22239109

**Published:** 2022-11-24

**Authors:** Justin A. Mahlberg, Howell Li, Björn Zachrisson, Dustin K. Leslie, Darcy M. Bullock

**Affiliations:** 1 Joint Transportation Research Program, Purdue University, West Lafayette, IN 47907, USA; 2 NIRA Dynamics AB, 58330 Linköping, Sweden; 3 Indiana Department of Transportation, West Lafayette, IN 47906, USA

**Keywords:** connected vehicles, crowdsourced data, inertial laser profiler, international roughness index (IRI), pavement, pavement quality

## Abstract

Modern vehicles have extensive instrumentation that can be used to actively assess the condition of infrastructure such as pavement markings, signs, and pavement smoothness. Currently, pavement condition evaluations are performed by state and federal officials typically using the industry standard of the International Roughness Index (IRI) or visual inspections. This paper looks at the use of on-board sensors integrated in Original Equipment Manufacturer (OEM) connected vehicles to obtain crowdsource estimates of ride quality using the International Rough Index (IRI). This paper presents a case study where over 112 km (70 mi) of Interstate-65 in Indiana were assessed, utilizing both an inertial profiler and connected production vehicle data. By comparing the inertial profiler to crowdsourced connected vehicle data, there was a linear correlation with an R^2^ of 0.79 and a *p*-value of <0.001. Although there are no published standards for using connected vehicle roughness data to evaluate pavement quality, these results suggest that connected vehicle roughness data is a viable tool for network level monitoring of pavement quality.

## 1. Introduction

The United States has over 266.9 million automobiles and trucks, and over 14.2 million lane-kilometers (8.8 million lane-miles) nationwide that require preservation [1]. In 2019, federal spending for highways was $46 billion. The recent highway bill allocates $110 billion in additional funding for road and bridge repair [2,3]. With over six million kilometers (four million miles) of roads in the United States, it is a difficult task to prioritize road segments and allocate resources targeting the segments that need repair the most [4,5,6]. Current methods for evaluating road quality involve performing inspection with specialized equipment to assess pavement condition and ride quality [7,8,9,10,11]. Some studies have used image processing techniques to estimate IRI and detect potholes [12,13,14,15,16,17]. Recent initiatives use LiDAR for pavement inspection because it can provide additional information on roadway drainage, pavement markings, and lane widths [18,19,20,21,22,23,24]. The limitations of these methods are that they often require instrumentation of a dedicated vehicle and an employee to drive the road network.

**Figure 1:**
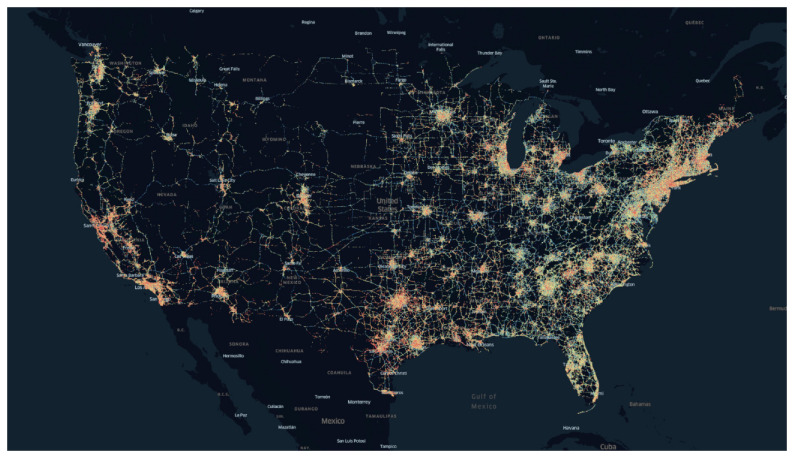
Current connected production vehicle coverage across the United States.

An alternative to dedicated equipment is to crowdsource connected vehicle data with enhanced attributes. Original Equipment Manufacturers (OEM) integrate enhanced sensors, accelerometers, and cellular connections into production vehicles to provide a rich data source on current road conditions. Integrating and utilizing the enhanced vehicle data have provided agencies with pavement marking evaluations, lane widths, traffic signal performance, and crash mitigation through surrogate safety measures and hard braking [25,26,27,28,29,30]. A recent invention leverages individual wheel speed via rotational sensors in combination with drivetrain information to provide pavement quality information in production vehicles [31]. This information can estimate International Roughness Index (IRI) values from a fleet of crowdsourced vehicles. Figure 1 below shows the connected vehicle coverage across the US using anonymized, enhanced production vehicle data for pavement quality evaluation in May of 2022. 

## 2. Motivation

**Figure 2:**
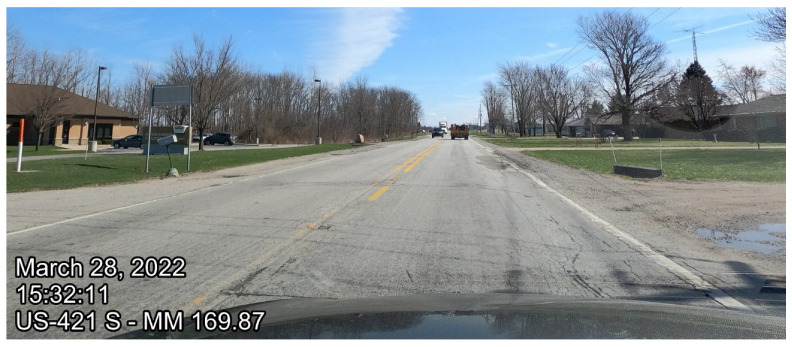
US-421 pavement condition in Delphi, Indiana.

Current means of evaluation of pavement conditions often include estimation of the International Roughness Index (IRI) with an inertial profiler. IRI is widely employed due to its stability overtime and has been applied around the world [32,33,34]. IRI is less subjective than other pavement performance indicators as it is calculated from the road profile. This method requires the agency to annually inspect all their roadways to determine maintenance procedures and budgets. Statewide evaluation, prioritization of segments, and maintenance is difficult to achieve within one season [7]. This costly procedure often results in agencies only conducting detailed data collection on their busiest roads, with lower-volume roads receiving less attention (Figure 2). 

## 3. Objectives

The objective of this study is to determine if pavement roughness measurements from crowdsourced sensors onboard production vehicles can be used to screen public road networks for sections that have less than the prescribed IRI. The benefit of using crowdsourced data from production vehicles is that it can provide ubiquitous coverage without additional vehicle instrumentation and reduce resource and personnel-intensive deployments. 

## 4. Data Collection Equipment and Methods

Ground-truth data were collected with:

- a calibrated inertial profiler;
- a single production vehicle; and
- GoPro cameras for pavement roughness validation.

**Figure 3:**
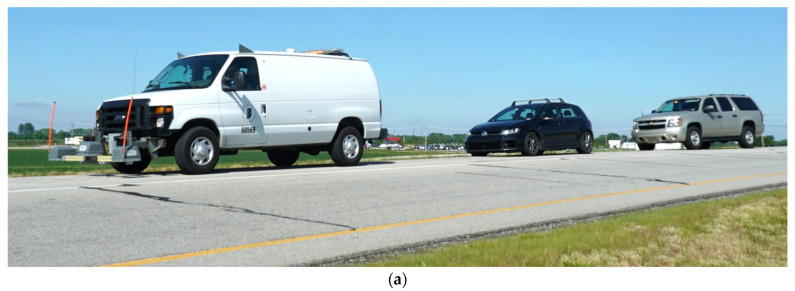

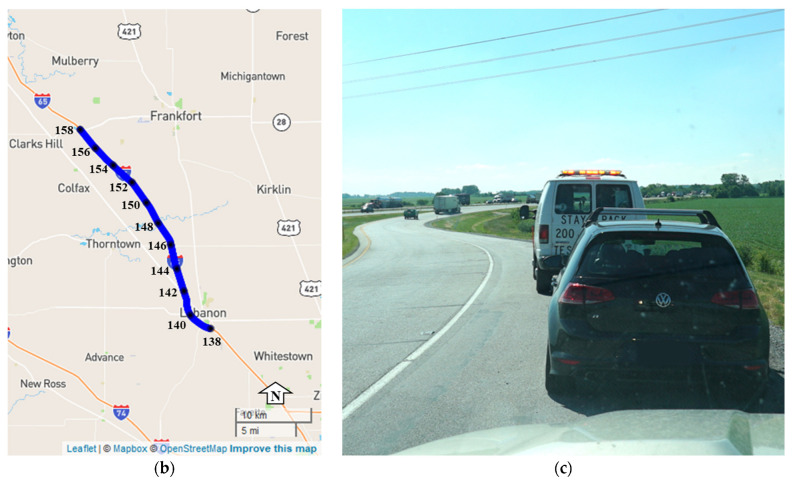
Data collection route and equipment; (**a**) data collection convoy—lead: inertial profiler, second: production vehicle, final: safety vehicle with hazard lights; (**b**) Indiana I-65 from reference marker 138 to 158; (**c**) data collection convoy.

For the purposes of this study, the vehicles were run in tandem, seen in Figure 3a,c, on the same lane and with similar wheel paths. The data collection route (Figure 3b) is Interstate 65 near Lebanon, Indiana from reference marker 138 to reference marker 158 in the north, and southbound directions in the left and right lane, respectively, with a distance of 32 km. These reference markers aid the agency to quantify sections of roadways for maintenance. Locally, they are also referred to as mile-markers (mm). The route also includes a 12 km (eight-mile) construction zone in both directions, with varying pavement quality and pavement material, which is why this section was chosen for analysis.

**Figure 4:**
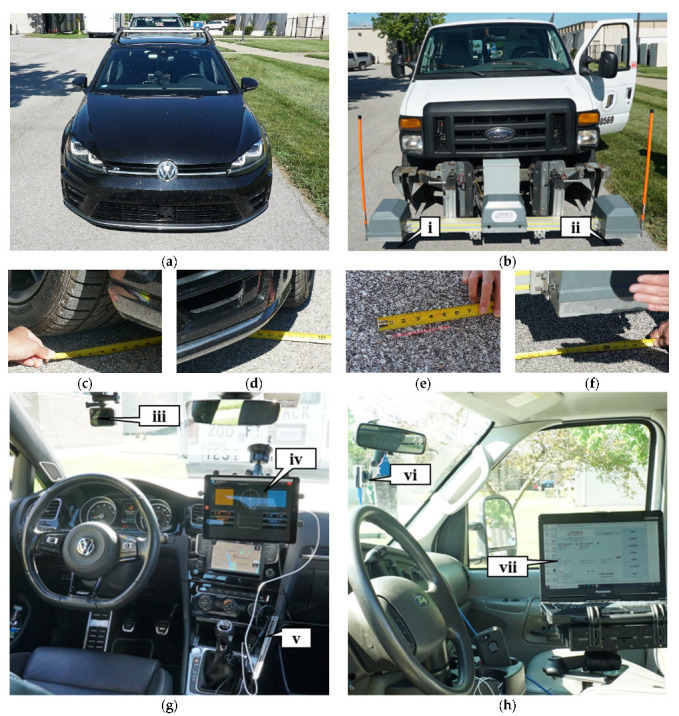
Production vehicle and profiler data collection equipment: (**a**) production vehicle; (**b**) inertial profiler; (**c**) production vehicle track width (passenger side); (**d**) production vehicle track width (driver side); (**e**) profiler laser width (passenger side); (**f**) profiler laser width (driver side); (**g**) interior of production vehicle; (**h**) interior of inertial profiler. Callout (i) laser scanner (passenger side); (ii) laser scanner (driver side); (iii) GoPro camera in production vehicle; (iv) user interface (production vehicle); (v) laptop data logger; (vi) dash camera (inertial profiler); (vii) user interface (inertial profiler).

There are several important components for data collection. The production vehicle (Figure 4a) determines pavement roughness through individual wheel-speed signals and sensors in the drivetrain to produce an IRI value. The inertial profiler seen in Figure 4b tabulates roughness using the vehicle’s speed, accelerometer, and laser scanners to determine the height to the pavement (callout i and ii) [35]. The profiler must be moving and have a minimum operating speed of 24 kph (15 mph) and a maximum of 96 kph (60 mph), which is why the vehicles captured data near the maximum threshold with minimal impact to traffic at 88 kph (55 mph). The measurements for both vehicles are dependent on the wheel path. Due to this dependency, the track width for the production vehicle and the laser width on the profiler were measured. Figure 4c shows the end of the tape measure at the passenger side of the connected production vehicle, and Figure 4d shows the driver side of the vehicle and the track width is approximately 178 cm (70 in). Figure 4e shows the end of the tape measure on the passenger side of the profiler vehicle and Figure 4f shows the driver side of the vehicle and the width of measurement is approximately 185 cm (73 in). This measurement provides confidence that if similar trajectories are taken by the vehicles, they will be scanning the same surface. For qualitative validation of pavement conditions, both vehicles are fitted with dash cameras. Figure 4g shows the interior of the production vehicle setup. Callout iii shows the GoPro camera that captures images at half-second frequency, callout iv shows the user interface while logging roughness data, and callout v shows the laptop that is connected to the data logger to collect the data. Figure 4h shows the interior of the profiler, callout vi shows the dash camera, which takes images every 30 m (100 ft), and callout vii shows the user interface.

The team logged data on Interstate 65 near Lebanon shown in Figure 3b, first in the left lane southbound direction, then the left lane northbound direction, followed by the right lane southbound direction, and lastly the right lane in the northbound direction. The profile data produces IRI values every 3 m (10 ft), and the production vehicle produced IRI values every 0.02 s. Due to the difference in data collection frequency, both the profile data and production vehicle were mapped to the nearest 0.01-reference marker on Interstate 65, which is approximately 16 m (53 ft). This led to an average of 333 IRI values per kilometer for the inertial profiler and about 2051 IRI values per kilometer for the production vehicle.

## 5. Single Production Vehicle Pavement Roughness Data vs. Inertial Profiler

**Figure 5:**
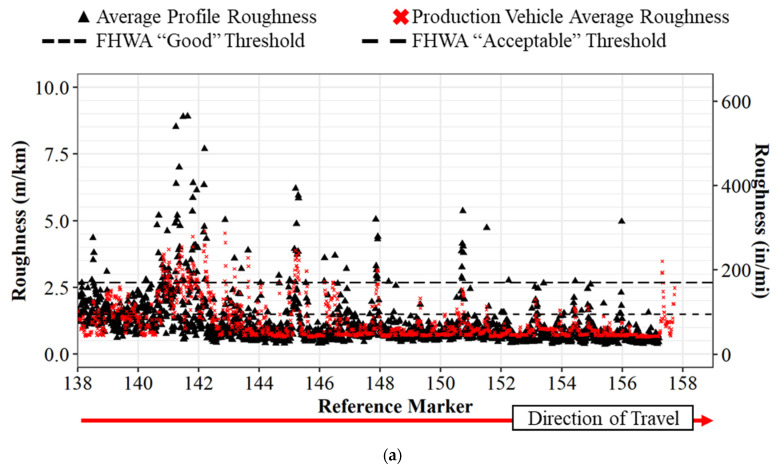

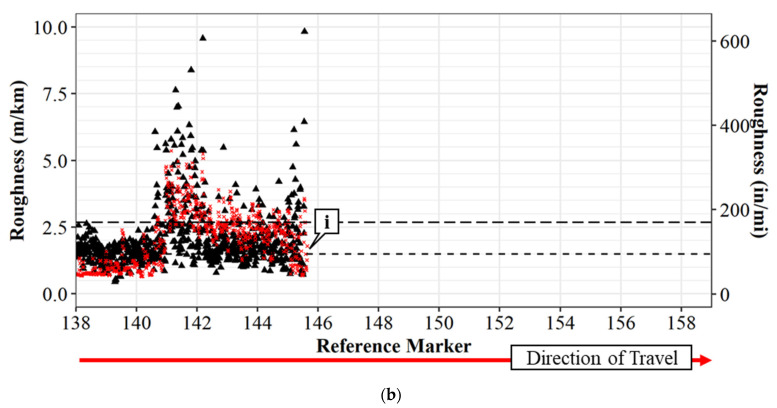
IRI comparison between inertial profiler and single production vehicle for I-65 northbound: (**a**) left lane; (**b**) right lane. Callout (i) data collection discontinued due to congestion.

**Figure 6:**
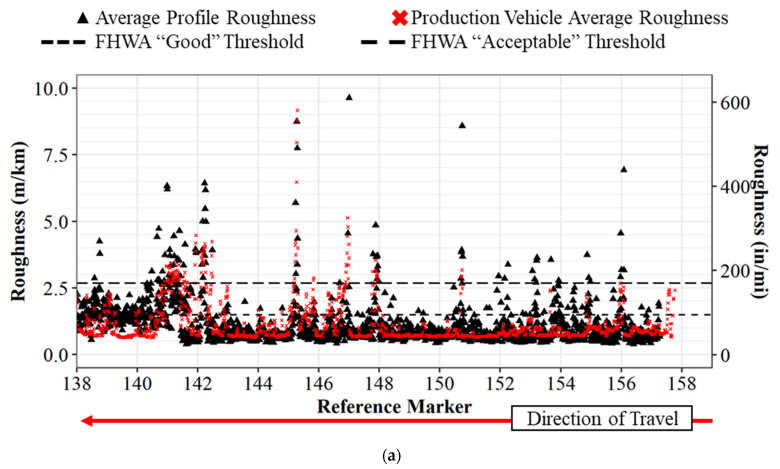

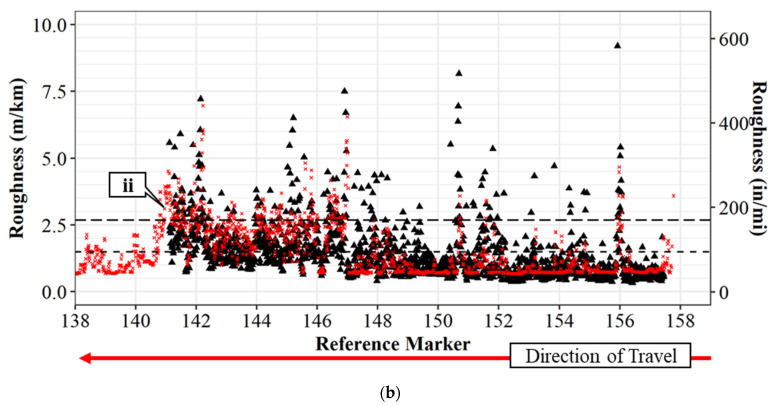
IRI comparison between inertial profiler and single production vehicle for I-65 southbound: (**a**) left lane; (**b**) right lane. Callout (ii) location where the profile data is suspended due to file size limitations.

The raw roughness values for Interstate 65 northbound can be seen in Figure 5 below, where the profiler values are in black, and the production vehicle values are red. The horizontal axis shows the reference marker on the interstate, and the vertical axis shows the IRI roughness. There are also two horizontal dotted lines at 1.49 m/km (95 in/mi) and 2.68 m/km (170 in/mi). These lines represent the national standard in the US for IRI thresholds. IRI values less than 1.49 m/km (95 in/mi) are classified as “good” road segments, whereas values below 2.68 m/km (170 in/mi) are “acceptable” road segments [36]. Values above 2.68 m/km (170 in/mi) surpass the threshold and require strategic repairs, maintenance, and rehabilitation. These thresholds set by the Federal Highway Administration (FHWA) also align with rider comfort [37]. Figure 5a shows the roughness values for the left lane and Figure 5b shows the roughness values for the right lane. At callout i in Figure 5b, the data collection was discontinued for both vehicles due to congestion on the roadway leading to speeds less than 24 kph (15 mph), which is below the operating threshold for the inertial profiler. Due to the difficulty in scheduling the inertial profiler, as the vehicle collects a large quantity of pavement evaluation for the state of Indiana, the team was not able to repeat the data collection. A similar comparison can be seen for Figure 6 in the southbound direction. Callout ii in Figure 6b refers to the point where the profile data is suspended due to file-size limitations. At a glance, the trend between the standard profiler values and the production readings are quite comparable at the same point in the roadway. The raw data from the production vehicle mapped to the inertial profiler data with a linear regression R^2^ value of 0.7493 on 6278 data points and a *p*-value < 0.001. These results suggest good agreement between the IRI and the inertial profiler IRI. 

## 6. Crowd-Sourced Pavement Roughness Data vs. Inertial Profiler

Although a single vehicle could be driven by agency personnel around the state to replicate the standard practice with production vehicles, a much more scalable approach would be to passively receive this data from a fleet of vehicles on a daily basis. Roughness data derived from production vehicle sensors provide a robust system for agencies to collect quantitative pavement quality information. Such an approach would allow an agency to have a dynamic, crowdsourced view of how their pavement quality changes over time.

**Figure 7:**
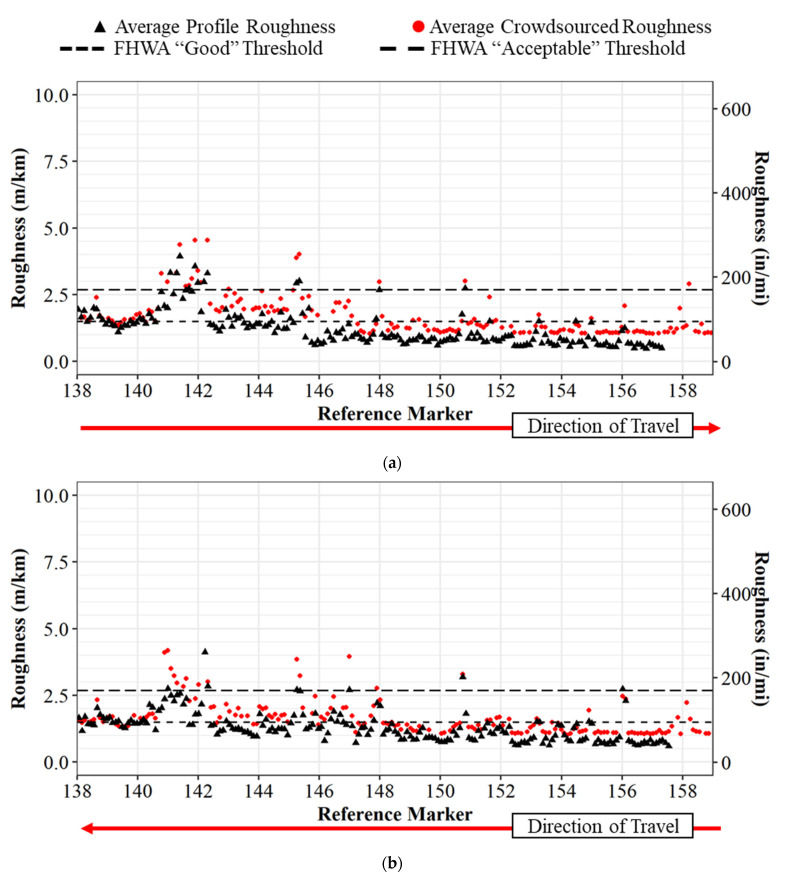
IRI comparison between inertial profiler and crowdsourced roughness for I-65: (**a**) northbound; (**b**) southbound.

A comparison of the crowdsourced roughness data (red) and the inertial profiler data (black) is shown in Figure 7 below. This comparison is linearized and referenced to the nearest 0.16 km (0.1 mi) segment on Interstate 65 as the crowdsourced data are provided in 22 m (75 ft) segments with no lane-level fidelity with current fleet models. Due to the lack of lane location, the profile data are linearized to the nearest 0.16 km (0.1 mi). Both the left and right lane are also combined for an average roughness. Figure 7a shows the northbound crowdsourced roughness and profiler roughness, and Figure 7b shows the southbound direction.

**Figure 8:**
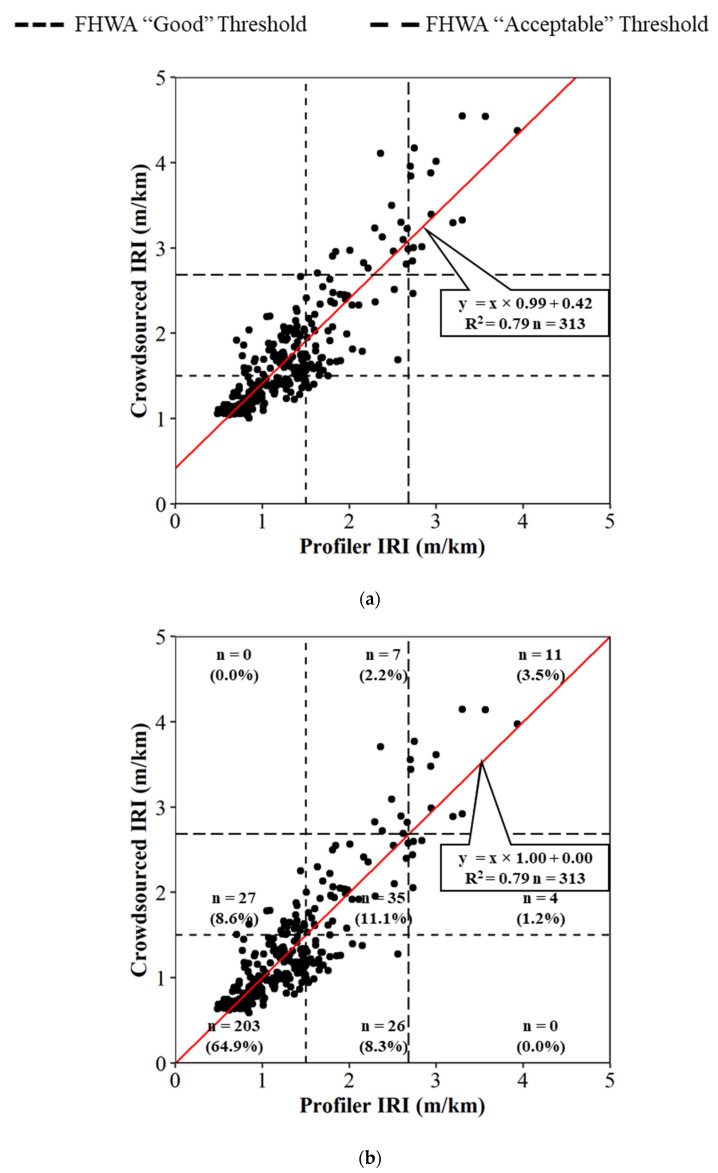
Linear correlation of profile IRI and crowdsourced IRI: (**a**) original data; (**b**) transformed crowdsourced data.

The crowdsourced data tracks the profiler data well for both the northbound and southbound directions. To provide a statistical evaluation of this relationship, average profiler IRI and crowdsourced production vehicle IRI values for each 0.16 km (0.1 mile) section is plotted in a scatter plot (Figure 8a). A linear trend-line is then drawn over the data points with an R2 of 0.79. A Pearson test was performed and a *p*-value of less than 0.001 suggests there is statistical significance to reject the null hypothesis of no linear correlation. Based on the linear trendline, there seemed to be a systematic offset of the production vehicle data being greater than the profiler data, as observed in Figure 7 and from the trendline in Figure 8a. The difference can be attributed to how each technology estimates IRI, and a more objective comparison of the methods, potentially with manual hand instruments, is suggested for future research. The current research conforms the production vehicle measurements to the laser profiler by applying an offset of 0.41 m/km (26 in/mi). The transformed linear correlation can be seen in Figure 8b. The equation used to transform the data is in Equation (1):

Transformed Crowdsourced IRI = Crowdsourced IRI − 0.42(1)

The same national standard IRI thresholds from the previous plots are also shown in Figure 8 (dotted lines). These lines make nine quadrants on the linear correlation graph and can help determine the accuracy of the crowdsourced roughness values compared to the profiler roughness. Looking at Figure 8b after the transformation, there are 203 averaged points (32.6 km or 20.3 mi) that are considered to be good road segments in both the crowdsourced data and the profiler data. There are also 35 points (5.6 km or 3.5 mi) that are acceptable for both data sets, and 11 points (1.8 km or 1.1 mi) that are outside the threshold for both datasets. The crowdsourced production data provided the same classification as the profiler data for 80% of all 0.16 km (0.1 mi) segments collected.

**Figure 9:**
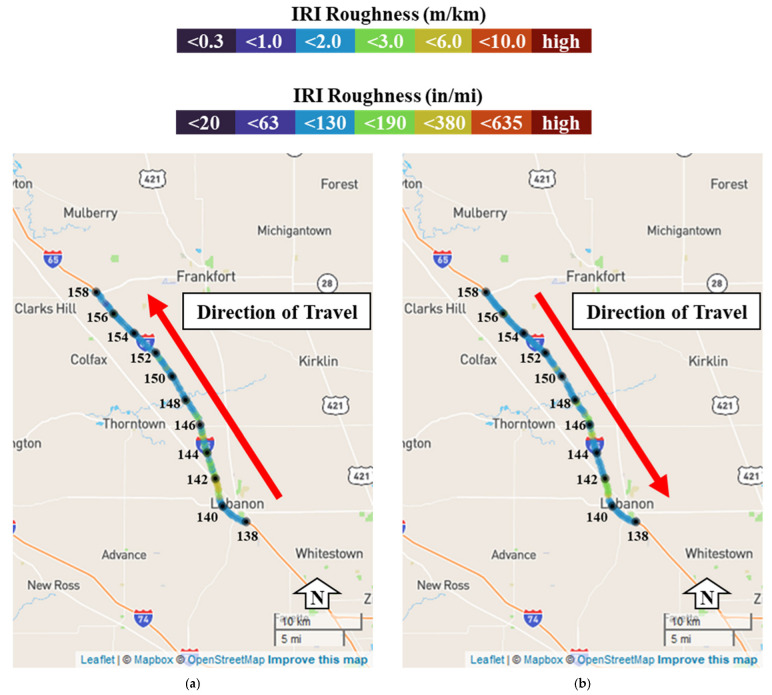
Spatial crowdsourced pavement roughness for I-65: (**a**) I-65 northbound; (**b**) I-65 southbound.

To provide more context and provide agencies with a tool to determine areas of pavement that require attention, the crowdsourced production vehicle roughness is plotted as a spatial map in Figure 9 below. Figure 9a shows the IRI roughness of Interstate 65 northbound colored by roughness, and Figure 9b shows the IRI roughness of Interstate 65 southbound. This geographical presentation of the data provides agencies with a tool to effectively identify problematic areas. An example of this can be observed from reference markers 141 to 142 in Figure 9 where the values are at or above 3.0 m/km (190 in/mi). This same trend was shown as a scatter plot in Figure 7.

## 7. Results and Discussion

Scheduling inertial profiler runs requires considerable planning and coordination to perform a run on a specific roadway. This is an expensive process and does not scale well for statewide monitoring of pavement condition on a real-time basis. In contrast, data from crowdsourced production vehicles are available within 24 h, providing aggregated roughness values for roadway segments estimated by multiple vehicle-passes over the segment in the previous 60 days. Uncertainty values are provided for each segment, as different vehicles may take different wheel paths through the system, e.g., avoidance of potholes, and may produce different results in the short-term. The advantage to crowdsourced data is agencies no longer must collect or process the data. They can analyze the crowdsourced data and efficiently prioritize maintenance activities.

### 7.1. Prioritizing Maintenance

**Figure 10:**
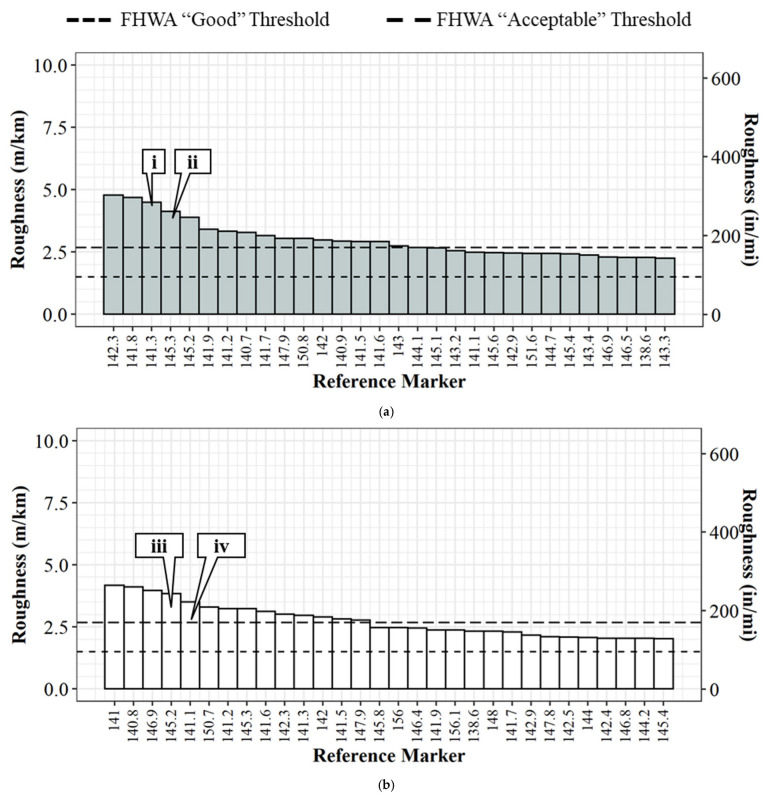
I-65 pavement roughness on I-65 from reference marker 138 to 158 in rank order, highest thirty values: (**a**) northbound; (**b**) southbound. Callout (i) Figure 11a; (ii) Figure 11b; (iii) Figure 11c; (iv) Figure 11d.

**Figure 11:**
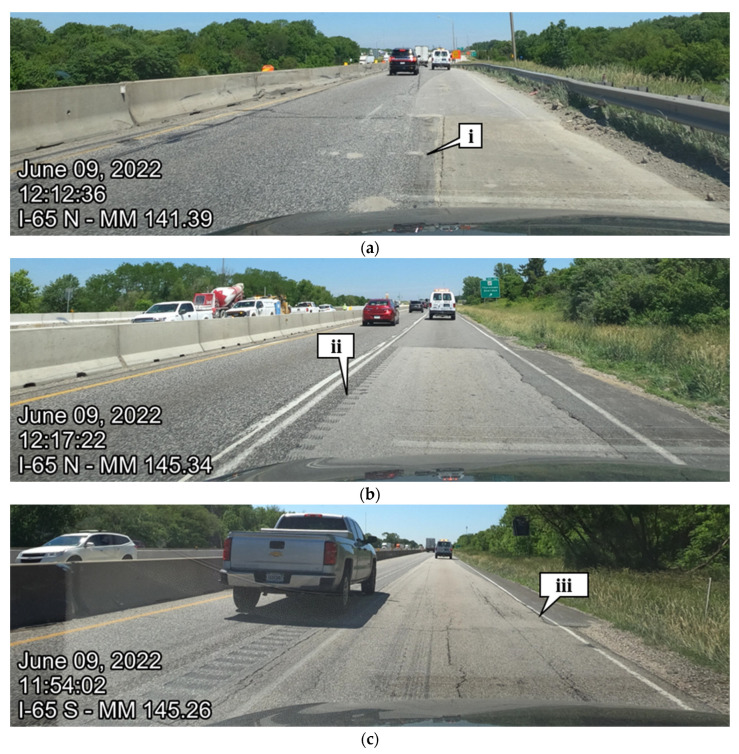

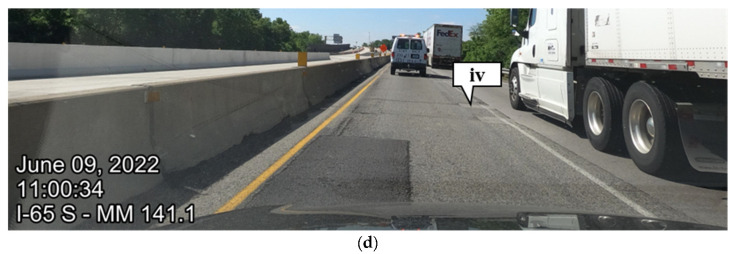
I-65 pavement distress validation points: (**a**) I-65 northbound reference marker 141.3; (**b**) I-65 northbound reference marker 145.3; (**c**) I-65 southbound reference marker 145.3; (**d**) I-65 southbound reference marker 141.1. Callout (i) pavement spotting; (ii) rumble strips in driving lane; (iii) depression in pavement; (iv) pothole.

Crowdsourced data for the 32 km (20 mi) of the interstate provides agencies with an efficient tool for determining where there is poor road quality in that section. Using this data, an agency can pareto-sort the average roughness by 0.16 km (0.1 mi) segments to determine segments that need maintenance the most. Figure 10a below shows the 30 highest roughness values pareto-sorted (rank order) on Interstate 65 northbound. It can be quickly noted that reference markers 142.3, 141.8, 141.3, 145.2, and 141.9 have the highest roughness values in the northbound direction. Callouts i and ii can be seen qualitatively in Figure 11a,b, respectively. Figure 10b shows a pareto-sorted graph for the five segments with the highest roughness are 141.0, 140.8, 146.9, 145.2, and 141.1 for Interstate 65 in the southbound direction. Figure 11c,d are representative images of callouts iii and iv, respectively. 

Figure 11a shows pavement spotting on Interstate 65 northbound reference marker 141.3 (Figure 10 callout i), Figure 11b shows rumble strips in the driving lane on I-65 northbound reference marker 145.3 (Figure 10 callout ii), Figure 11c shows a depression in the pavement on Interstate 65 southbound reference marker 145.2 (Figure 10, callout iii), and Figure 11d shows a pothole on Interstate 65 southbound reference marker 141.1 (Figure 10, callout iv). All four examples are located within the construction zone boundaries.

### 7.2. Pavement Improvement Evaluation after Reconstruction

**Figure 12:**
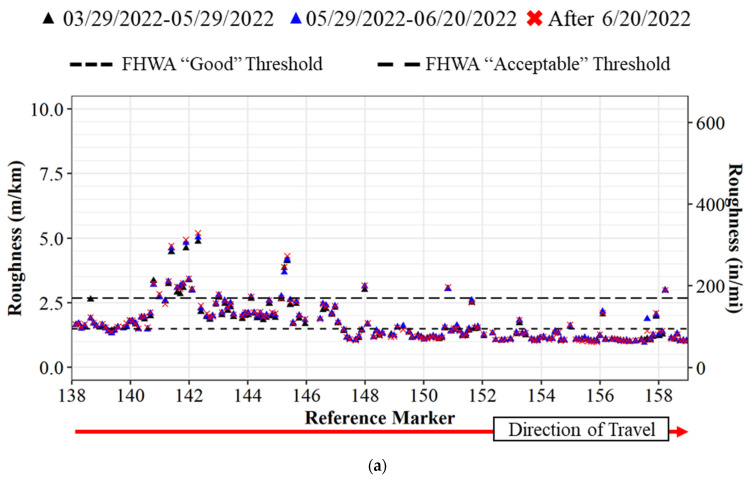

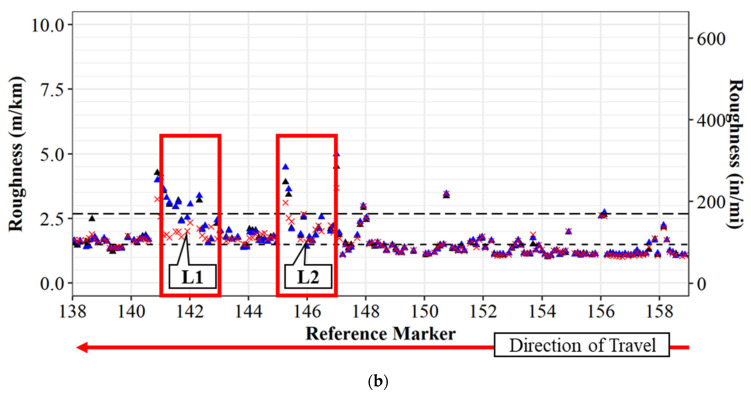
IRI comparison on I-65 from March 2022 to July 2022: (**a**) I-65 northbound 3-month analysis; (**b**) I-65 southbound 3-month analysis. Callout (L1) reference marker 142 shown in Figure 13a,b; (L2) reference marker 146 shown in Figure 13c,d.

**Figure 13:**
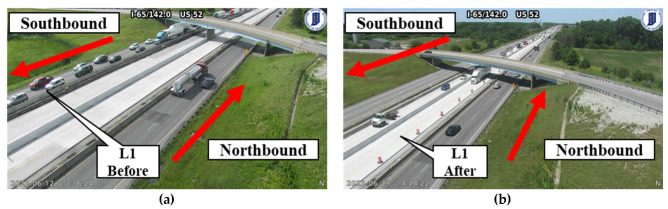

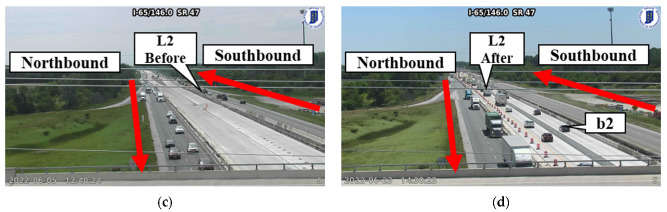
I-65 traffic cameras for roughness validation: (**a**) reference marker 142.0 before 20 June 2022; (**b**) reference marker 142.0 after 20 June 2022; (**c**) reference marker 146.0 before 20 June 2022; (**d**) reference marker 146.0 after 20 June 2022.

Another advantage to the crowdsourced data is that it would provide agencies with a way to track pavement deterioration over time. Figure 12 is an example of pavement roughness before a complete reconstruction of the pavement and after traffic is moved to the new pavement in the southbound direction on 20 June 2022. It can be noted that there is almost no change in the roughness for the northbound traffic (Figure 12a) as there is no change in traffic patterns. For the southbound direction, there is a change in roughness after 20 June 2022. Traffic is moved to the new pavement and the roughness decreases. Due to the aggregation of the crowdsource data, the roughness for each segment is a 60-day rolling average, and any previous measurements would require 60 days to clear out. The transition from old to new pavement can be verified using Indiana Department of Transportation traffic cameras at callouts L1 and L2 in Figure 13 below.

Figure 13a shows location L1 from Figure 12b. This camera is at reference marker 142 and the image was taken on 12 June 2022. It can be noticed that the northbound traffic is operating in the far rightmost lanes and the southbound traffic is operating in the leftmost lanes. Figure 13b shows the same location on 23 June 2022, where the southbound traffic is now operating in the middle two lanes of newly constructed pavement. Figure 13c is at reference marker 146 (callout L2 in Figure 12b) and the camera is facing the opposite direction from Figure 13a,b. In this image, the northbound traffic is operating in the two leftmost lanes and the southbound traffic is operating in the rightmost lanes on 5 June 2022. Figure 13d shows the southbound traffic operating on the new pavement in the center two lanes on 23 June 2022. 

### 7.3. Network Level Pavement Quality Assessment

**Figure 14:**
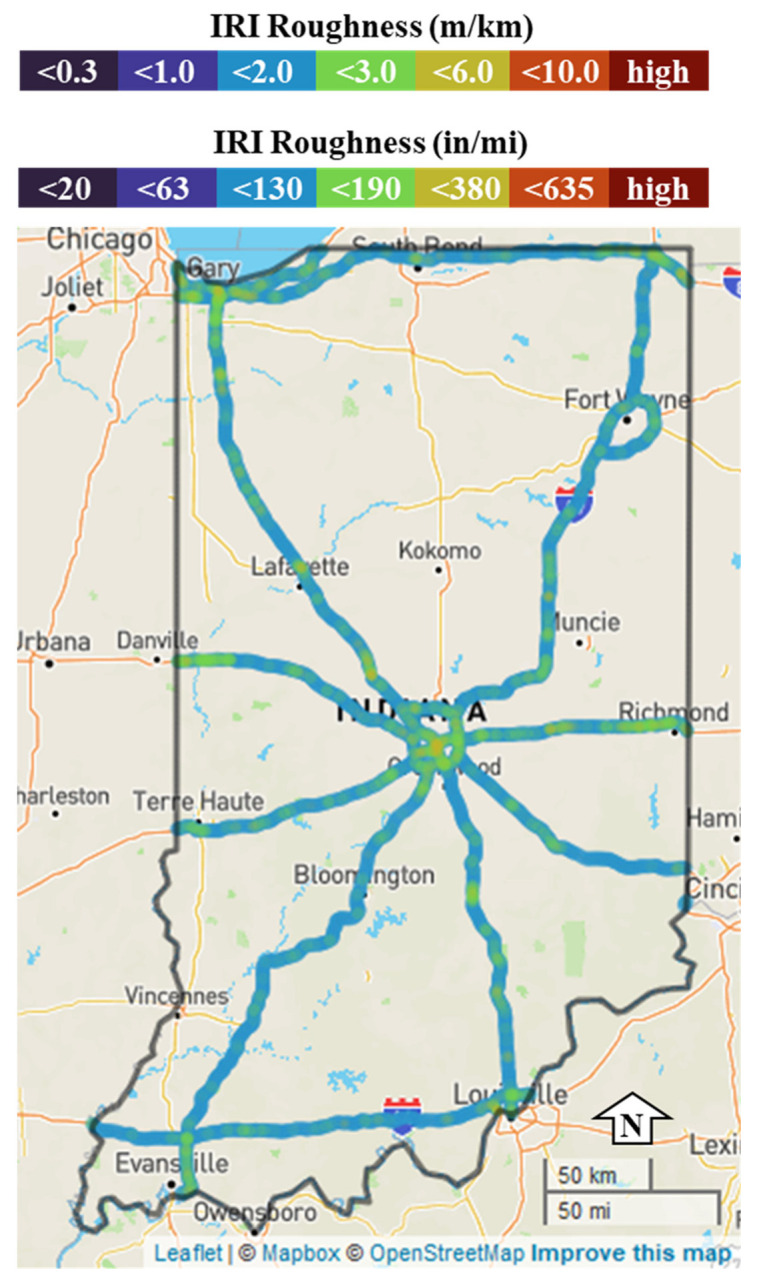
Statewide pavement roughness.

The greatest advantage of crowdsourced data is the ability to evaluate, assess, and prioritize infrastructure maintenance without having to allocate the time, resources, or equipment to gather the information. This data provides accurate real-time pavement conditions that is redundant and repeatable. Figure 14 shows statewide average IRI values on all Indiana interstates. This information provides agencies the tools necessary to prioritize road segments that need maintenance and other locations that are performing well. Such an approach would allow an agency to have a dynamic, crowdsourced view of how their pavement quality changes over time. 

## 8. Conclusions and Future Scope

Traditional methods of collecting pavement roughness data with a single vehicle inertial profiler are difficult to economically scale system-wide and repeat on an ongoing basis due to the required time to collect and process data. This paper demonstrated both quantitatively and qualitatively how connected vehicle data provides an opportunity for dynamic and scalable operational assessment of pavement quality. Three datasets, including the inertial profiler data, a single production vehicle’s data, and a crowdsourced dataset, were utilized to determine that connected production vehicle data can provide accurate pavement roughness values and classifications. The analysis revealed there were similar trends between the profiler and a single production vehicle (Figure 5 and Figure 6) and that there is a linear correlation between crowdsourced data and the profiler data (Figure 8) with an R2 of 0.79 and a *p*-value of <0.001.

This analysis provides evidence that connected vehicle crowdsourced roughness data can be utilized for network-wide analysis. The availability of data nationwide and ease of scalability makes this data implementable on a state or national basis for tracking any highway pavement quality without equipment being deployed. These techniques can provide a nationwide opportunity to revisit current guidelines for pavement quality evaluation and engage in conversations on how design procedures may be expanded to incorporate new datasets for the next generation of pavement-maintenance needs assessment.

## Data Availability

Not applicable.
